# Wildflower Strips Promote Spider Diversity and Biological Control Potential in a Semi-Arid Agroecosystem: Preliminary Insights from a Single Growing Season

**DOI:** 10.3390/insects17070722

**Published:** 2026-07-13

**Authors:** Meilan Tuo, Wenchao Hu, Dongyang Wu, Weifu Liu, Shuting Wang, Xingren Long, Yajun Jin

**Affiliations:** All-College of Biological Science and Engineering, North Minzu University, 204 Wenchang North Street, Xixia District, Yinchuan 750021, China

**Keywords:** wildflower strips, farmland spiders, spatio-temporal dynamics, edge effect, temporal refuge, Yinchuan plain, ecological pest control

## Abstract

Agricultural intensification degrades farmland biodiversity, prompting the use of wildflower strips to restore natural enemy populations. However, their spatio-temporal effects on predators in arid agroecosystems remain unclear. Therefore, we investigated ground-dwelling spiders in sown wildflower strips versus natural margins across four cropping systems in arid northwestern China using pitfall traps along a spatial gradient throughout the growing season. While the overall diversity did not differ significantly between treatments, three key patterns emerged: First, spider abundance remained stable across the spatial gradient, whereas taxonomic diversity increased significantly toward the crop interior. Second, while communities in natural margins peaked early and declined during the hot, dry mid-summer, wildflower strips maintained large populations, acting as crucial temporal refuges. Third, wildflower strips reorganized species co-occurrence networks, fostering more resilient community structures. We conclude that in water-limited environments, wildflower strips stabilize predator populations during harsh weather and enhance community resilience. These findings highlight the value of incorporating these strips when designing sustainable ecological pest management strategies for arid agroecosystems.

## Simple Summary

Intensive farming negatively impacts biodiversity and natural pest control. While planting wildflower strips along field edges helps to increase the prevalence of beneficial predators such as spiders, the effectiveness of these strips in dry regions over time and in different areas remains unclear. We studied how wildflower strips affect ground-dwelling spiders across four crop types in arid northwestern China throughout a growing season and found that while total spider numbers were similar between wildflower strips and natural edges, the strips maintained large spider populations during the hot, dry mid-summer when natural edges experienced a sharp decline. Furthermore, spider diversity increased further into the crop fields, and the strips reorganized how spider species interact, allowing a more resilient community to form. We conclude that in dry environments, wildflower strips are valuable tools for stabilizing predator populations during harsh weather. This knowledge will help farmers to design better ecological pest control strategies, reducing their reliance on chemical pesticides and promoting sustainable food production in water-limited regions.

## 1. Introduction

Global agricultural intensification has delivered substantial gains in food production, but these gains have come at a considerable ecological cost. Monoculture cropping, habitat fragmentation, and excessive agrochemical inputs have degraded farmland ecosystem functioning and substantially reduced biodiversity and the ecosystem services it sustains, including pollination and natural pest control [1,2,3]. Losses of natural enemy-mediated pest suppression in intensified agricultural landscapes have been estimated at over 40%, while global insect abundance has declined by nearly 50% over the past four decades [4]. Nature-based solutions (NbSs) have been identified as a key response to these challenges. Within this framework, establishing ecological infrastructure—such as vegetated buffer strips, ecological ditches, and wildflower strips (WFSs)—at field margins or within cropped areas has been identified as a central strategy for restoring functional diversity in agroecosystems [5], as re-establishing predator–prey dynamics appears critical to the recovery of ecosystem services [6]. To counteract the negative impacts of agricultural intensification, habitat diversification strategies, particularly the implementation of sown wildflower strips (WFSs), have been widely adopted to restore biodiversity and ecosystem functioning [7,8]. The literature on flowering strips is highly abundant, spanning more than three decades. Since the early 1990s, numerous studies, particularly originating from Europe, have extensively investigated their ecological functions and structural benefits in intensive agricultural landscapes [9,10,11]. It is now well established that increasing vegetation complexity within these landscapes significantly affects a broad range of arthropod taxa. Extensive research has demonstrated that WFSs not only benefit spiders but also provide crucial shelter, alternative prey, and floral resources for diverse beneficial insect communities, including ground beetles (*Carabidae*), rove beetles (*Staphylinidae*), parasitoids, predatory insects (such as green lacewings), and pollinators [12,13,14].

Among these natural enemies, ground-dwelling spiders (*Araneae*) are highly abundant generalist predators that play a pivotal role in conservation biological control [15]. The implementation of grassy field margins and WFSs has been proven to foster spider abundance, species richness, and functional diversity across various farming systems [16]. More importantly, distinct spider species and diverse spider assemblages have demonstrated significant effectiveness against specific agricultural pests. For example, specific spider communities have been well-documented as effective biological control agents capable of suppressing populations of major pests, such as aphids in cereal crops [17], leafhoppers in agricultural fields [18], and specific lepidopteran larvae in orchards [19]. By maintaining robust spider communities through habitat management, agroecosystems can benefit from their complementary hunting strategies and temporal niche partitioning, which ultimately improves the overall suppression of specific pest outbreaks [20,21,22].

Wildflower strips (WFSs) provide a multidimensional ecological baseline within agricultural matrices [23]. By supplying nectar, pollen, alternative prey, and structurally heterogeneous microhabitats, they support the persistence of diverse natural enemies [24]. Positive population responses have been well documented among parasitic wasps [25], lacewings [26], ladybirds [27], and carabid beetles [28]. Orchard-based interventions in the UK, for example, have driven substantial increases in spider and parasitoid abundance [29], while field experiments in Germany have demonstrated enhanced spider body-size differentiation and functional spillover effects [16].

This body of evidence, however, suffers from an important methodological limitation, in that previous studies have predominantly relied on cross-sectional snapshots of biodiversity metrics [30,31], whereas spatial gradients of dispersal decay and temporal successional trajectories have been insufficiently characterized. Real-world ecological radiation from marginal habitats into intensive farmland is rarely uniform; rather, it is modulated by edge effects, species colonization capacity, and phenological asynchrony across seasons [32,33]. Whether the “stable increases” observed in existing studies represent genuine restoration of agroecosystem resilience or transient aggregation within a particular spatial–temporal window has yet to be determined. Moreover, empirical validations of WFSs are heavily biased toward temperate and semi-humid environments [34]; their applicability to arid agroecosystems—where physiological constraints imposed by moisture deficits and temperature variability differ on a fundamental level—has received comparatively little attention [35].

Spiders constitute one of the most abundant and functionally diverse groups of invertebrate predators in agroecosystems, employing foraging strategies (cursorial, web-building, and ambush) that play a critical role in suppressing pest outbreaks [36,37]. In contrast to highly mobile parasitoids, ground-dwelling spiders have limited dispersal ability and are therefore particularly sensitive to fine-scale habitat configurations and seasonal environmental fluctuations [38]. Non-crop habitats, such as WFSs, can serve both as permanent refugia and as “temporal refuges” during periods of agricultural disturbance or elevated environmental stress [39]. Landscape and local habitat attributes also shape spiders’ taxonomic composition [40]. Nevertheless, the mechanisms by which WFSs restructure the spatial distribution of farmland spider assemblages and buffer seasonal fluctuations remain insufficiently quantified.

The Yinchuan Plain anchors high-yield agriculture within China’s arid and semi-arid Northwest. The region is characterized by large-scale monoculture cropping compounded by ongoing urbanization, which has substantially simplified the native habitat matrix [41]. Under these conditions, the behavioral ecology of epigeal predator communities is likely shaped by physiological constraints imposed by moisture deficits and large diurnal temperature fluctuations. Whether floral marginalia retain their proposed buffering capacity in such dryland settings and what spatio-temporal dynamics underlie their ecological function remain open questions.

We addressed these questions through a field experiment embedding symmetrical configurations of engineered WFSs and bare-margin controls across four dominant cropping architectures (wheat, maize, tomato, and apple orchard). Ground-dwelling spider assemblages were monitored continuously via pitfall trapping across a full growing season. This study pursued three specific objectives: (i) to quantify total spider abundance, species richness, and Shannon diversity in WFSs versus control margins, both overall and stratified by spatial position and month; (ii) to identify spatial gradients in assemblage composition and associated key taxa from margin edges into the crop interior; and (iii) to characterize the co-occurrence network structure and its response to WFS deployment, with a particular focus on shifts in keystone species roles across treatments.

## 2. Materials and Methods

### 2.1. Study Area

**Figure 1:**
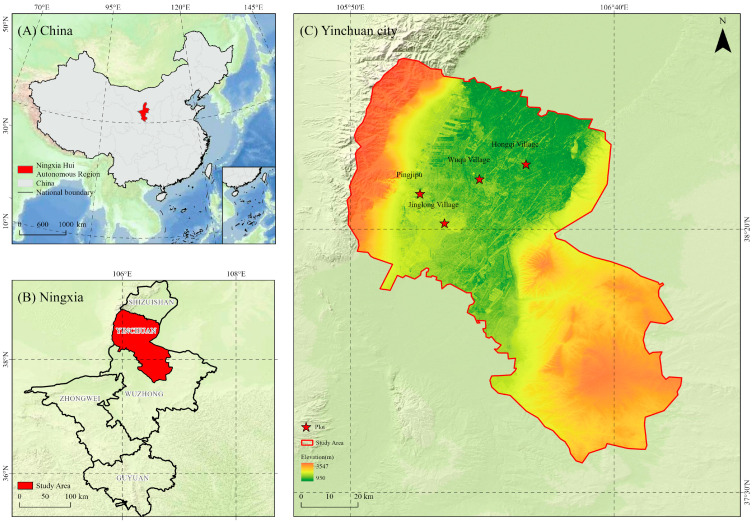
Geographic location of the study area and distribution of the sampling sites. (**A**) Map of China indicating the position of the Ningxia Hui Autonomous Region. (**B**) Map of Ningxia showing the location of Yinchuan City. (**C**) Detailed map of the study area in the Yinchuan Plain, illustrating the spatial distribution of the four experimental sampling sites across different cropping systems: Wuqu Village (wheat), Pingjipu (maize), Hongqi Village (processing tomato), and Jinglong Village (apple orchard). Scale bars are provided for spatial reference.

The study was conducted in the Yinchuan Plain (approximately 37°29′–38°53′ N, 105°49′–106°53′ E), a critical intensive agricultural zone in the arid to semi-arid transition of northwestern China (Figure 1). The region features a temperate continental climate characterized by high solar radiation, large diurnal temperature fluctuations, and a pronounced water deficit. Meteorological data from the nearest regional station indicate an average annual temperature of 8.5 °C, mean annual precipitation of 180–220 mm, and potential evapotranspiration exceeding 2000 mm. The frost-free period spans 150–170 days annually. Soils are predominantly alluvial fluvo-aquic soils with a light loam to sandy loam profile and pH ranging from 8.2 to 8.4.

Agricultural production in this region is heavily reliant on Yellow River irrigation and is predominantly dominated by large-scale monocultures of spring wheat (*Triticum aestivum* L.), maize (*Zea mays* L.), processing tomato (*Solanum lycopersicum* L.), and apple orchards (*Malus domestica* Borkh.). To evaluate the efficacy of ecological infrastructure under these arid agroecological constraints, we selected four representative commercial fields across Helan County and Liangtian Town. The specific geographical coordinates of the four experimental sites are as follows: Wuqu Village (wheat field; 38.4920° N, 106.2413° E), Pingjipu (maize field; 38.4457° N, 106.0533° E), Hongqi Village (processing tomato field; 38.5393° N, 106.3882° E), and Jinglong Village (apple orchard; 38.3524° N, 106.1306° E) (Figure 1). These sites were selected to ensure uniform baseline soil properties and representative regional management histories within each respective cropping system.

### 2.2. Meteorological Data and Climatic Context

**Figure 2:**
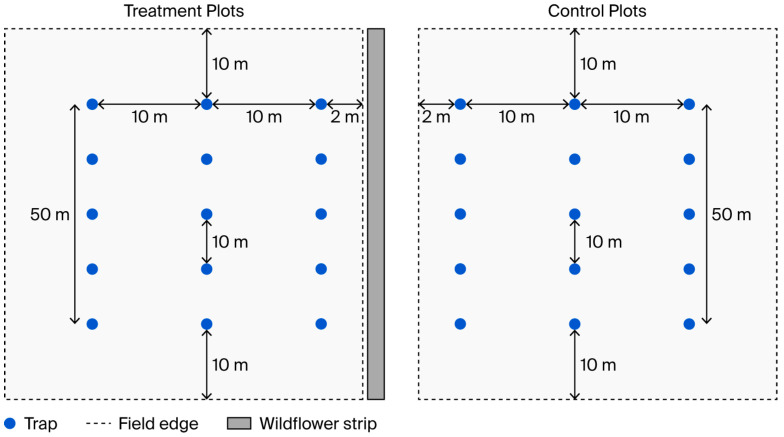
Experimental design and spatial layout of pitfall trapping arrays. Trap transects were deployed perpendicular to the crop–margin interface at three nominal distances (0 m, 10 m, and 20 m) into the crop field, across paired WFSs and control margin treatments and four cropping systems.

To contextualize the environmental conditions under which the spider assemblages were monitored, daily meteorological data—including mean temperature and precipitation—were obtained from the Global Surface Summary of the Day (GSOD) dataset provided by the National Oceanic and Atmospheric Administration (NOAA). Data were extracted from the Yinchuan Meteorological Station (WMO ID: 53614; 38.32° N, 106.39° E; elevation: 1141 m), the nearest standard regional station to the study area. To evaluate whether the single study year (2024) was representative of the regional climate, we compiled a 20-year historical dataset (2004–2023) from the same station to calculate long-term monthly averages for temperature and total precipitation. These data were used to assess the occurrence of any extreme meteorological events during the sampling period and to serve as a baseline for comparison (see Figure 2).

### 2.3. Experimental Site Characteristics and Agronomic Management

The four experimental fields varied in size from approximately 1.7 to 4.0 hectares. Prior to the establishment of the current cropping systems, all fields shared a consistent cropping history, predominantly rotating between maize and wheat over the preceding five years, with no prior implementation of conservation biological control practices (e.g., sown floral margins). The specific crop species and commercially cultivated varieties at each site were: spring wheat (cv. Ningchun 4) at Wuqu Village; maize (cv. Xianyu 335) at Pingjipu; processing tomato (cv. variety, e.g., Ningfan 8) at Hongqi Village; and apple (cv. variety, e.g., Red Fuji) at Jinglong Village.

Conventional agronomic management practices across all sites followed standard regional protocols for intensive production, which are known to significantly influence epigeal arthropod assemblages. Tillage regimes consisted of conventional intensive autumn deep plowing (25–30 cm depth) followed by spring rotary cultivation (10–15 cm depth) with crop residue incorporation, except for the apple orchard, which maintained permanent natural ground cover with periodic mechanical mowing. Irrigation was primarily dependent on the Yellow River water network, utilizing transitionary surface flood irrigation for wheat and maize, and drip irrigation for the tomato field and apple orchard.

To allow for proper ecological interpretation of the spider assemblage data, comprehensive details regarding the specific fertilization regimes (types and application rates of N, P, and K fertilizers), precise irrigation schedules, and the complete pesticide application records—including the active ingredients, commercial names, application rates, and exact timing of all synthetic insecticides, herbicides, and fungicides used at each site during the 2024 growing season—are provided in Table S1 (Supplementary Materials). Crucially, within each experimental block, the sown wildflower strips (WFSs) and the adjacent crop fields were strictly managed without any insecticide or herbicide applications on the margins themselves to preserve non-target natural enemy populations.

Although systematic quantitative vegetation surveys (e.g., Braun–Blanquet relevés) were not conducted due to the primary focus on predator assemblages, the botanical composition of the WFSs was explicitly designed to ensure continuous floral resource provisioning throughout the growing season based on established functional trait data for the selected species [42]. The annual herbs (*Calendula officinalis*, *Zinnia elegans*, *Cosmos bipinnatus*) provided high-intensity nectar and pollen resources from late May through September, covering the critical mid-summer period when natural margins typically senesce. Perennial and biennial components (*Medicago sativa*, *Melilotus officinalis*, *Astragalus adsurgens*) ensured early-season establishment and structural persistence into late autumn. This staggered phenological design was intended to maintain a continuous supply of floral resources and microhabitat complexity, thereby supporting the temporal refuge function for spider assemblages observed in this study. Detailed flowering phenology and functional traits of the sown species are provided in Table S2.

### 2.4. Experimental Design and Spider Sampling

We implemented a paired comparative design across four distinct cropping systems to evaluate the effects of sown wildflower strips (WFSs) against naturally regenerated bare margins (control). Within each of the four selected commercial fields, a WFS treatment was paired with an adjacent control plot, separated by a minimum 500 m buffer to prevent cross-treatment spillover. The sown wildflower strips (WFSs) were established in April 2023. The seedbed was prepared by shallow rotary tillage, followed by surface broadcasting of tailored seed mixtures and initial irrigation. Strips measured 3 m in width and 60 m in length, running continuously parallel to the crop field edge. Control margins were maintained under standard local farmer practice, permitting natural ruderal regeneration without sowing. These naturally regenerated margins were not bare soil; rather, they supported a sparse to moderate cover of locally adapted ruderal vegetation, predominantly comprising annual and perennial weeds typical of the Yinchuan Plain agroecosystem. The dominant ruderal species observed included *Chenopodium album* L., *Setaria viridis* (L.) P. Beauv., *Artemisia annua* L., *Convolvulus arvensis* L., and *Echinochloa crus-galli* (L.) P. Beauv., along with scattered individuals of *Sonchus oleraceus* L. and *Calystegia hederacea* Wall. The vegetation height in control margins typically ranged from 15 to 40 cm during the early growing season but progressively senesced during the hot, dry mid-summer period (July–August), resulting in reduced structural complexity and limited ground-level shading. Although these ruderal communities provided some degree of microhabitat structure and partial shade for ground-dwelling spiders during the early season, their phenological decline under peak thermal and hydric stress contrasted sharply with the sustained vegetation cover maintained in the sown wildflower strips.

These naturally regenerated margins were not bare soil; rather, they supported a sparse to moderate cover of locally adapted ruderal vegetation, predominantly comprising annual and perennial weeds typical of the Yinchuan Plain agroecosystem. The dominant ruderal species observed included *Chenopodium album* L., *Setaria viridis* (L.) P. Beauv., *Artemisia annua* L., *Convolvulus arvensis* L., and *Echinochloa crus-galli* (L.) P. Beauv., along with scattered individuals of *Sonchus oleraceus* L. and *Calystegia hederacea* Wall. The vegetation height in control margins typically ranged from 15 to 40 cm during the early growing season but progressively senesced during the hot, dry mid-summer period (July–August), resulting in reduced structural complexity and limited ground-level shading. Although these ruderal communities provided some degree of microhabitat structure and partial shade for ground-dwelling spiders during the early season, their phenological decline under peak thermal and hydric stress contrasted sharply with the sustained vegetation cover maintained in the sown wildflower strips.

The botanical composition of the WFSs was specifically tailored to the agronomic and ecological context of each cropping system (Table S2). Rather than applying a uniform seed mixture across all sites, we designed site-specific formulations to account for differences in crop architecture, management intensity, and local microclimatic conditions. Across the four sites, a total of 21 plant species were utilized, encompassing annual herbs, perennial herbs, biennials, and shrubs (Table S2).

At the Wuqu Village (wheat) and Pingjipu (maize) sites, the seed mixtures were dominated by annual flowering herbs (e.g., *Calendula officinalis*, *Zinnia elegans*, *Cosmos bipinnatus*, and *Centaurea cyanus*), selected for their rapid establishment, prolonged flowering phenology, and high nectar and pollen production, which are critical for sustaining diverse arthropod assemblages throughout the growing season. Perennial species such as *Astragalus adsurgens*, *Iris lactea*, and *Medicago sativa* were incorporated at the Pingjipu site to provide stable ground cover and overwintering refugia. At the Hongqi Village (tomato) site, the mixture was anchored by the biennial *Melilotus officinalis* (40%), supplemented with shrubs (*Sorbaria kirilowiiand*, *Rosa xanthina*) to provide permanent structural complexity, and *Tagetes erecta*, which is known for its nematode-suppressing properties. In contrast, the apple orchard at Jinglong Village utilized a monoculture of *Medicago sativa* (100%), a standard regional practice for orchard ground cover that provides nitrogen fixation, stable overwintering habitat, and continuous ground-level vegetation structure. This context-dependent botanical design ensures that the ecological infrastructure is functionally aligned with the specific pest–predator dynamics and structural requirements of each agroecosystem matrix.

Epigeal spider assemblages were monitored continuously from 10 May to 28 August 2024 using standardized pitfall trapping arrays. Within each block, trap transects were deployed perpendicular to the crop–margin interface at nominal distances of 0, 10, and 20 m into the crop field. To avoid microclimatic artifacts at the physical boundary, the “0 m” transect was deliberately offset 2 m into the crop interior. At each distance, five traps were installed as spatial replicates, spaced ≥5 m apart parallel to the margin to minimize spatial autocorrelation, and positioned ≥10 m from any lateral field boundaries. Traps consisted of 200 mL rigid plastic cups (7.5 cm internal diameter, 7.3 cm depth) buried flush with the soil surface, pre-filled with 100 mL of a 1:5 (*v*/*v*) propylene glycol–water solution amended with unscented surfactant. A waterproof plastic roof was suspended 3 cm above the opening to exclude rainfall and vertebrate bycatch. Traps were serviced at strict 6-day intervals, yielding 12 discrete sampling events. Specimens were preserved in 75% ethanol and identified to the species level under stereomicroscopes.

### 2.5. Calculation of Diversity Indices

To comprehensively evaluate the spider assemblages, both alpha and beta diversity metrics were calculated. Alpha diversity was assessed using four indices:

(1) Species diversity (H′) was evaluated using the Shannon–Wiener index:

$$H^{\prime }\;=-\sum \left(P_{i}\;\ln\;P_{i}\right)$$

where $P_{i}$ is the relative abundance of the i-th species.

(2) Species richness (C) was assessed using the Margalef richness index:

$$C\;=\;\frac{\left(S\;-\;1\right)}{\ln\;N}$$

where S  denotes the total number of species and N  is the total abundance.

(3) Species evenness (J) was determined using Pielou’s evenness index:

$$J\;=\;\frac{H^{\prime }}{\ln\;S}$$

(4) Species dominance (D) was analyzed using the Simpson dominance index:

$$D\;=\;1\;-\;\sum {\left(P_{i}\right)}^{2}.$$

Beta diversity, representing the turnover in community composition across treatments and temporal gradients, was quantified using the Bray–Curtis dissimilarity matrix, which served as the foundation for subsequent multivariate ordination analyses.

### 2.6. Data Analysis

Species identification was based on authoritative taxonomic references, including the Fauna Sinica [30] and the Ecological Atlas of Chinese Spiders [31]. All adult specimens were identified to the species level wherever possible. A small number of adult individuals that could not be confidently assigned to a known species were excluded from species-level analyses to maintain taxonomic rigor. Juvenile specimens were excluded from all subsequent analyses. This is a standard practice in arachnological studies, as juveniles cannot be reliably identified to the species level. Including them would introduce significant taxonomic uncertainty, thereby compromising the accuracy of diversity metrics (e.g., species richness, Shannon index) and community composition analyses, which are central to this study’s objectives. Our focus on adult spiders, the primary reproductive and predatory stage, ensures a robust assessment of the long-term effects of wildflower strips on the functional spider community.

Differences in alpha diversity metrics (abundance, richness, and Shannon index) between treatments (WFSs vs. Control) and across spatial distances (0, 10, and 20 m) were analyzed using Wilcoxon rank-sum tests. To align with the seasonal successional analysis, data from the six-day interval sampling events were aggregated into monthly totals (May, June, July, and August).

Non-metric Multidimensional Scaling (NMDS) ordination based on the Bray–Curtis dissimilarity matrix was performed to evaluate shifts in overall community composition (beta diversity) driven by margin treatments and seasonality. The 95% confidence ellipses and community centroids were plotted to visualize spatial segregation and temporal successional trajectories. Indicator Species Analysis (ISA) was performed using the point-biserial correlation coefficient to identify taxa strongly associated with specific spatial zones (e.g., field edge vs. crop interior).

Species co-occurrence networks were constructed based on Spearman rank correlation coefficients to evaluate community structural shifts and intra-guild interactions. Prior to network construction, rare species were filtered to minimize spurious correlations arising from zero-inflated data: species occurring in fewer than five sampling units (i.e., fewer than five trap-events across the entire study) were excluded from the network analysis, as their predominantly zero-valued abundance records can artificially inflate both positive and negative correlation coefficients in standard Spearman analyses. For the retained species, pairwise Spearman correlations were computed, and *p*-values were adjusted for multiple testing using the Benjamini–Hochberg False Discovery Rate (FDR) method to control the expected proportion of false positives among the thousands of simultaneous pairwise comparisons. To construct the networks, only statistically significant (FDR-adjusted *p* < 0.05) and strong positive correlations (Spearman’s |r| > 0.6) were retained. We focused on positive correlations in the primary network analysis because our principal aim was to identify co-occurrence modules structured by shared environmental filtering and potential facilitative interactions—patterns that are particularly informative for understanding how wildflower strips reorganize community assembly in arid environments. We acknowledge that negative correlations can convey ecologically meaningful information regarding competitive exclusion or intra-guild predation among spider taxa (see Discussion, Section 4.3); however, given the relatively modest sample size and the prevalence of rare species in our dataset, negative correlations were more susceptible to statistical instability and were therefore not included in the primary network visualization. Network topological properties, including modularity, node degree, and identification of hub/keystone taxa, were calculated using the *igraph* package. All statistical analyses, network constructions, and visualizations were performed using R version 4.6.1 (R Core Team, 2026), with packages including *vegan* for NMDS, *indicspecies* for ISA, and *igraph* for network topology. A PERMANOVA (999 permutations) was used to validate multivariate patterns.

## 3. Results

### 3.1. Taxonomic Composition and Species Abundance

After excluding unidentifiable juveniles, a total of 2521 individual adult spiders were retained for analysis across all sampling events, representing 22 species from 10 families (Table S3). Lycosidae was the most species-rich and abundant family, comprising seven species and 1123 individuals (44.5% of the total community). Gnaphosidae, Thomisidae, and Araneidae were also well represented (with three species each). The assemblage was dominated by two cursorial lycosids—*Pardosa astrigera* L. Koch, 1878) and *Arctosa stigmosa* (Lowe, 1852)—which collectively accounted for nearly half of all individuals. *P. astrigera* was the most ubiquitous (655 individuals; 26.0% relative abundance; and 73.3% occurrence frequency), followed by *A. stigmosa* (468 individuals; 18.6%; 60.8% frequency). A cohort of 11 subdominant species (including *Pardosa laura* Karsch, 1879, *Lycosa coelestis* L. Koch, 1878, *Gnaphosa kansuensis* Schenkel, 1936, and *Xysticus hedini* Schenkel, 1936) exhibited moderate abundances (71–91 individuals). The remaining nine species (e.g., *Neoscona scylla* (Karsch, 1879), *Agelena labyrinthica* (Clerck, 1757)) were relatively rare.

Descriptive comparisons revealed distinct species-specific treatment responses. Dominant agrobionts like *P. astrigera* (375 vs. 280 individuals) and *X. hedini* (47 vs. 24) exhibited notably higher abundances in WFS plots. Conversely, specific subdominant taxa appeared more prevalent in naturally regenerated control margins, including *Lycosa sinesis* (34 vs. 47) and *Xysticus pseudoblitea* (24 vs. 47), indicating varying microhabitat preferences.

### 3.2. Climatic Conditions During the Study Period

**Figure 3:**
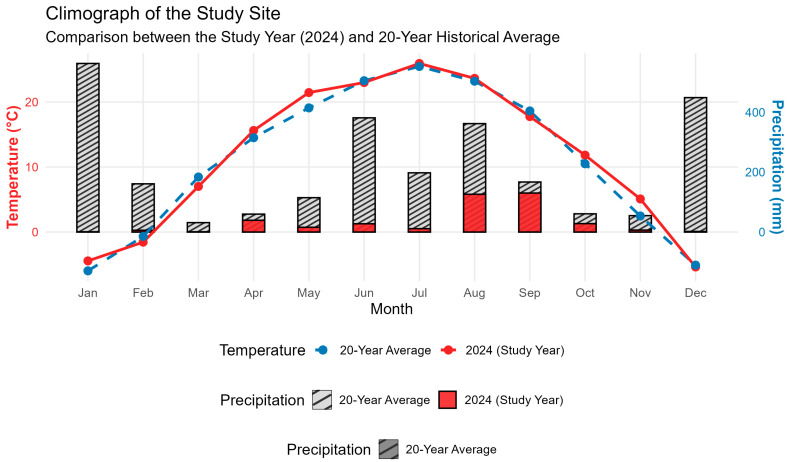
Climatic comparison between the study year (2024) and the 20-year historical baseline (2004–2023) at the study site in the Yinchuan Plain. Data were sourced from the Yinchuan Meteorological Station (NOAA GSOD, WMO ID: 53614). The left y-axis shows mean monthly temperature (°C), with the solid red line representing 2024 and the dashed blue line representing the 20-year historical average. The right y-axis shows total monthly precipitation (mm), with solid red bars for 2024 and hatched bars for the historical average. The above-average precipitation during the critical sampling period (May–August) is noted, while temperature patterns remained consistent with historical averages, confirming the general representativeness of the study year.

During the sampling period (May to August 2024), the study area experienced meteorological conditions that were highly representative of the typical semi-arid climate of the Yinchuan Plain in terms of temperature patterns. The monthly mean temperatures ranged from 12.4 °C in May to 24.7 °C in July, closely aligning with the 20-year historical averages (Figure 3). However, a notable deviation occurred in precipitation patterns: total precipitation during the four-month sampling window was 287.5 mm, which was 18.3% higher than the long-term average of 243.0 mm. Crucially, this increase was primarily driven by significantly higher rainfall in July (142.8 mm) and August (87.3 mm) compared to historical averages (July: 98.6 mm; August: 62.1 mm). This above-average precipitation during the critical sampling months may have enhanced vegetation growth in both wildflower strips and control plots, potentially increasing prey availability for spiders. Nevertheless, the absence of extreme temperature events and the moderate nature of the precipitation anomaly confirm that the observed spatio-temporal dynamics of the spider assemblages reflect typical ecological responses rather than artifacts of an extreme meteorological year.

### 3.3. Overall Effects of Wildflower Strips on Spider Alpha Diversity

**Figure 4:**
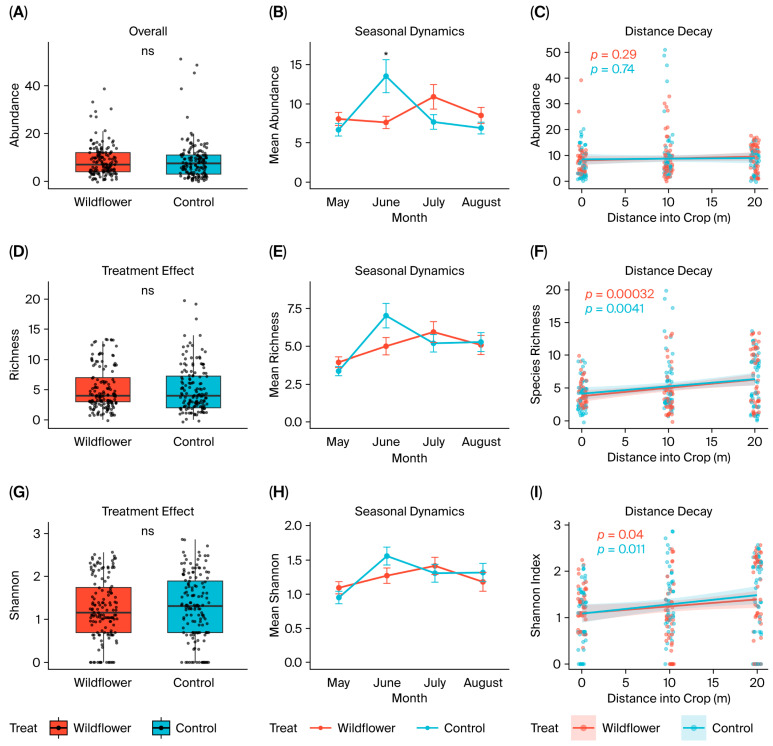
Spatio-temporal dynamics of ground-dwelling spider alpha diversity in WFS and control margins, encompassing overall aggregated effects (**A**,**D**,**G**), temporal trajectories across sampling months (**B**,**E**,**H**), and spatial gradients from the field edge to the crop interior (**C**,**F**,**I**). Statistical differences between treatments were evaluated using Wilcoxon rank-sum tests; spatial and temporal variations used Kruskal–Wallis with post hoc Dunn’s tests.

When data were aggregated across all sampling dates and spatial distances, the main effect of sown WFSs on overall alpha diversity metrics was not statistically significant compared to natural control margins. Boxplot analyses revealed no significant differences between treatments in terms of total spider abundance (Wilcoxon rank-sum test, *p* > 0.05, Figure 4A), species richness (*p* > 0.05, Figure 4D), or Shannon diversity index (*p* > 0.05, Figure 4G).

The observed increase in precipitation during July and August 2024 may have influenced spider assemblage composition. Specifically, the higher moisture levels likely promoted the growth of herbaceous vegetation in wildflower strips, creating more complex microhabitats that supported greater spider diversity. This is consistent with our finding that web-building spiders (primarily Linyphiidae and Theridiidae), which are often more sensitive to humidity conditions, showed particularly strong positive responses to wildflower strips (Figure 3 and Figure 4B,E,H). The enhanced vegetation structure in wildflower strips under wetter conditions may have provided additional foraging and shelter opportunities, thereby amplifying the beneficial effects on spider diversity compared to drier years. This suggests that wildflower strips may provide ecosystem services across a range of precipitation conditions, with potentially enhanced benefits in wetter years.

### 3.4. Context-Dependent Spatial Heterogeneity Across Cropping Systems and Distance Gradients

**Figure 5:**
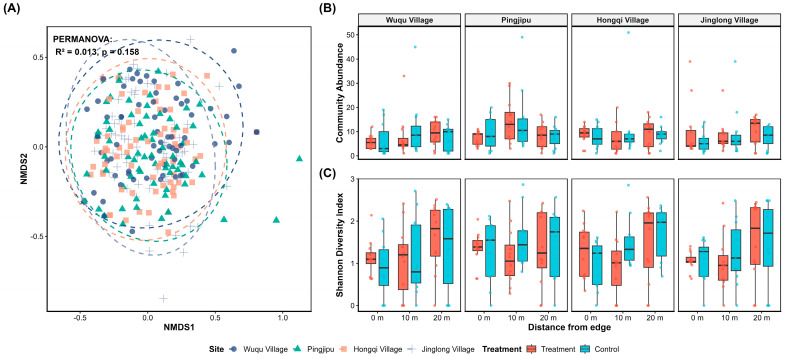
Context-dependent spatial heterogeneity of spider assemblages across four cropping systems and distance gradients. (**A**) Non-metric Multidimensional Scaling (NMDS) ordination of spider community composition (beta diversity). Colors represent the four sampling sites (cropping systems), with 95% confidence ellipses indicating group variation. Shapes denote treatment types (circles: wildflower strips; squares: control), showing extensive overlap within sites. (**B**) Boxplots depicting total spider abundance across the spatial gradient (0, 10, and 20 m from the field edge) stratified by sampling sites. (**C**) Boxplots depicting Shannon diversity index across the same gradients. Outliers are shown as individual points; box hinges represent the 25th and 75th percentiles, with the center line indicating the median. Statistical significance of the gradients was assessed by the Kruskal–Wallis test (*p* < 0.05).

While the aggregated analyses (Section 3.2) indicated no overarching main effect of WFSs on alpha diversity, multivariate ordination revealed that spider assemblages were profoundly influenced by local site conditions and cropping system contexts. NMDS ordination based on the Bray–Curtis dissimilarity matrix demonstrated distinct spatial segregation among the four sampling sites (Figure 5A). The 95% confidence ellipses for each site exhibited minimal overlap, indicating that the fundamental taxonomic composition of the ground-dwelling spider community was highly site-specific, likely driven by the distinct crop architectures (wheat, maize, tomato, and apple orchard) and localized microhabitat conditions. Notably, within each site-specific cluster, the geometric shapes representing WFSs (circles) and control (squares) treatments showed extensive overlap, corroborating the PERMANOVA results indicating that margin treatment alone did not lead to a drastic shift in the overarching beta diversity compared to the strong environmental filtering imposed by the cropping system.

This context dependency was further reflected in the univariate alpha diversity metrics stratified by site and distance into the crop field (Figure 5B,C). Total spider abundance exhibited pronounced site-specific variations, with certain cropping systems supporting substantially higher predator densities than others, yet the within-site response to the distance gradient (0, 10, and 20 m) remained highly heterogeneous and non-linear across the four locations. Similarly, the Shannon diversity index varied significantly among the four sites, underscoring that the baseline biodiversity pool and the subsequent spillover dynamics from the margin into the crop interior are fundamentally modulated by the specific agroecosystem matrix rather than operating as a uniform, landscape-wide response.

### 3.5. Spatial Gradients Dictate Biodiversity Rather than Abundance

Despite the absence of an aggregated treatment effect, spatial analysis (from 0 m to 20 m into the crop) revealed strong decoupling of abundance and diversity. Overall spider abundance remained relatively stable, exhibiting no significant response to distance in either the WFS (*p* = 0.29) or control (*p* = 0.74) treatments (Figure 4C). In contrast, taxonomic diversity exhibited a highly significant positive edge-to-interior gradient. Species richness increased significantly toward the crop interior (20 m) in both WFS (*p* = 0.0003) and control (*p* = 0.004) plots (Figure 4F). Similarly, the Shannon diversity index showed a significant positive correlation with distance into the field (WFS: *p* = 0.04; Control: *p* = 0.011; Figure 4I). This indicates that the crop interior inherently supports a significantly more diverse spider assemblage than the immediate edge.

### 3.6. Divergent Seasonal Successional Trajectories

Temporal monitoring revealed distinct seasonal dynamics between the two margin managements. In the control plots, all community metrics (abundance, richness, and Shannon index) exhibited a pronounced early-season peak in June, followed by a steep decline during July and August (Figure 4B,E,H). Conversely, spider communities associated with WFS demonstrated a delayed and buffered phenological trajectory: rather than peaking in June and subsequently declining, abundance and diversity following WFS treatments were sustained throughout July, when control plots had already entered a period of low activity (Figure 4E,H). This temporal divergence resulted in significant differences in seasonal abundance trajectories between treatments (Figure 4B, *p* < 0.05).

The divergence coincides with the period in which regional meteorological station data indicate maximum air temperatures and minimum rainfall in the Yinchuan Plain. However, in the absence of in situ microclimatic profiling, the extent to which WFS directly buffered spiders against environmental stress—versus merely supporting divergent phenological dynamics—has yet to be empirically validated.

### 3.7. Taxonomic Drivers of Spatial Gradients: Interior-Associated Indicator Species

**Figure 6:**
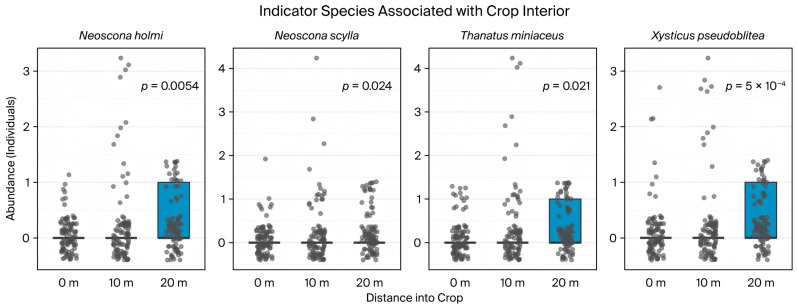
Results from Indicator Species Analysis (ISA) identifying specific taxa strongly associated with the crop interior (10 m and 20 m zones). Four significant indicator species (*Neoscona scylla*, *Neoscona holmi*, *Thanatus miniaceus*, and *Xysticus pseudoblitea*) are shown with edge-avoidance behavior from 0 m to 20 m. Indicator values (IndVal) and corresponding *p*-values are annotated for each taxon.

To identify the specific taxa driving the observed spatial patterns, we conducted an Indicator Species Analysis (ISA). This multivariate method objectively identifies species that are both faithful (high frequency of occurrence) and specific (high abundance) to a particular habitat type or environmental condition, making it a robust approach for selecting potential bioindicators. The analysis identified four taxa—*Neoscona scylla*, *Neoscona holmi*, *Thanatus miniaceus*, and *Xysticus pseudoblitea*—as highly significant indicator species for the crop interior (10 m and 20 m zones). As illustrated in Figure 6, these taxa exhibited pronounced edge-avoidance behavior. At the immediate field boundary (0 m), their populations were sparse, with median abundances near zero. However, a substantial colonization shift occurred at 20 m into the crop field, where the abundance of all four taxa increased significantly. This spatial partitioning was robust across all identified indicators, with high indicator values (IndVal): *X. pseudoblitea* (stat = 0.426, *p* = 0.003), *N. scylla* (stat = 0.425, *p* = 0.008), *T. miniaceus* (stat = 0.481, *p* = 0.008), and *N. holmi* (stat = 0.430, *p* = 0.008).

### 3.8. Species-Specific Temporal Dynamics of Lycosa Coelestis

**Figure 7:**
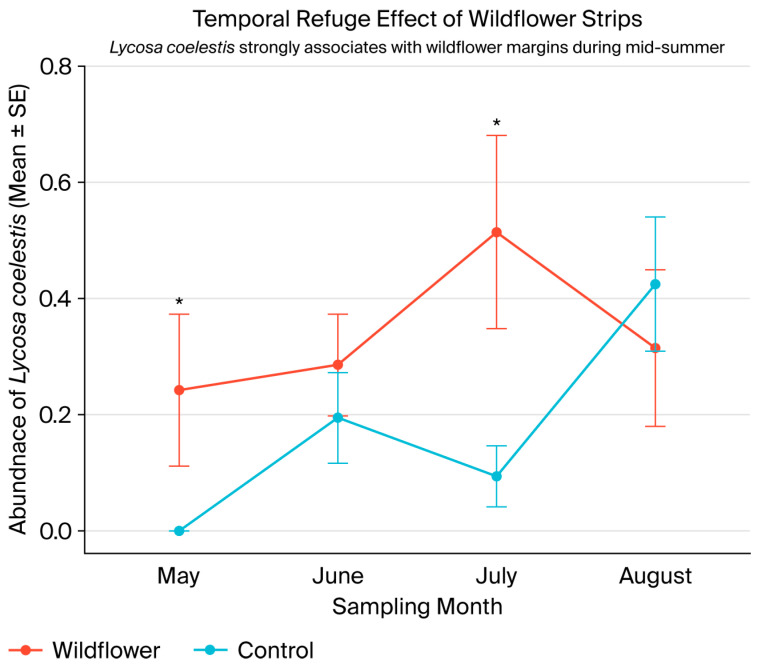
Species-specific temporal dynamics of *Lycosa coelestis* abundance in WFS and control margins across sampling months. Data are presented as mean SE. Statistically significant divergences between treatments were evaluated using Wilcoxon rank-sum tests and are denoted by asterisks (* *p*< 0.05).

While the overall community analysis revealed general trends, examining species-specific responses provides deeper mechanistic insights. We focused on *Lycosa coelestis* because it served as the most compelling example of the “temporal refuge” effect provided by WFSs. Among the dominant spider species, its temporal dynamics showed the most pronounced and statistically significant divergence between treatments during the critical mid-summer period. In contrast, other abundant species, such as the ubiquitous *Pardosa astrigera*, did not exhibit such a clear treatment-dependent response in July, highlighting the unique sensitivity of L. coelestis to the refuge conditions. The temporal abundance trajectory of L. coelestis revealed distinct, treatment-dependent seasonal patterns (Figure 7). Early in the season (May), *L. coelestis* established higher initial populations in WFS margins, whereas it was virtually absent in naturally regenerated control plots (Wilcoxon rank-sum test, W = 665.0, *p* = 0.0183). During June, abundances remained relatively low and comparable between the two treatments (*p* > 0.05). The most pronounced divergence occurred in July; while *L. coelestis* abundance remained persistently low in the control margins, its population exhibited a distinct and significant peak within the WFS (W = 674.5, *p* = 0.0394). By late summer (August), as populations in the control margins eventually increased, abundance converged between the two treatments and the statistical difference was no longer significant (W = 502.5, *p* = 0.2361).

### 3.9. Beta Diversity and Parallel Seasonal Successional Trajectories

**Figure 8:**
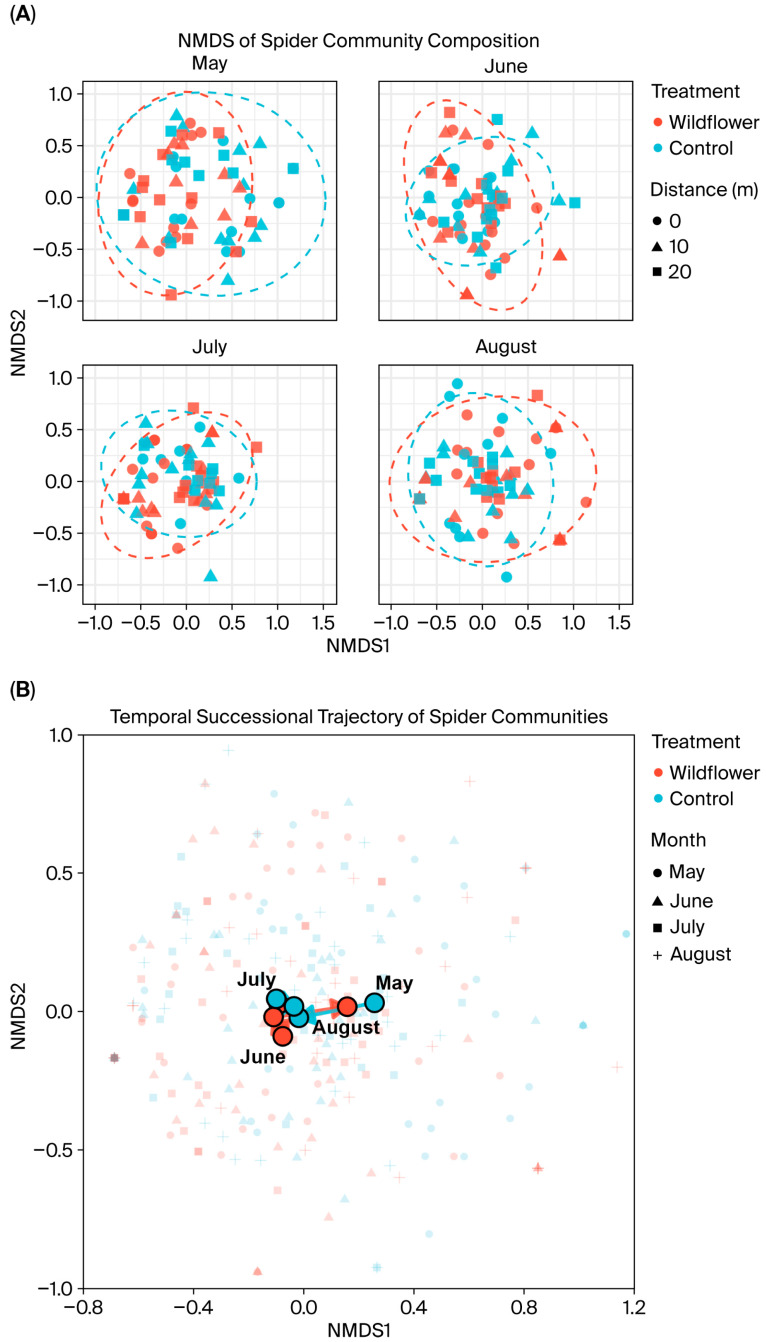
Beta diversity and seasonal successional trajectories of spider assemblages based on NMDS. (**A**) NMDS ordination faceted by sampling month (stress = 0.2219). (**B**) Temporal successional trajectories of community centroids from May to August.

NMDS ordination based on the Bray–Curtis dissimilarity matrix (stress = 0.2219) revealed that, when the community data were faceted by month (Figure 8A), the 95% confidence ellipses for the WFS and control treatments exhibited extensive and consistent overlap across all four sampling periods. This structural overlap indicates that the overarching multivariate composition of the spider assemblages was broadly similar between the two margin types at any given point during the season.

The analysis of community centroids over time revealed a clear temporal successional trajectory (Figure 8B), in which communities early in the season (May) clustered distinctly on the positive side of the NMDS1 axis. As the season progressed into June and July, the centroids shifted substantially toward the negative end of NMDS1, before slightly rebounding in late summer (August).

PERMANOVA (999 permutations) provided quantitative validation. The main effect of margin treatment (WFS vs. control) was not statistically significant (R^2^ = 0.004, *p* = 0.354), corroborating the visual overlap in the NMDS plot. However, community assembly was strongly driven by distance from the margin (R^2^ = 0.016, *p* = 0.001) and sampling month (R^2^ = 0.021, *p* = 0.003).

Importantly, while the successional vectors for WFS and naturally regenerated control communities moved in near-parallel synchrony, the treatment × month interaction was highly significant (R^2^ = 0.017, *p* = 0.008). This interaction suggests that the ecological difference between WFS and control margins is season-dependent, with pronounced divergence during mid-summer. Furthermore, the significant distance × month interaction (R^2^ = 0.041, *p* = 0.001) and three-way interaction (treatment × distance × month; R^2^ = 0.028, *p* = 0.022) highlighted that predator spillover from marginal strips into the crop interior dynamically fluctuated across the growing season.

### 3.10. Fundamental Rewiring of Species Co-Occurrence Networks

**Figure 9:**
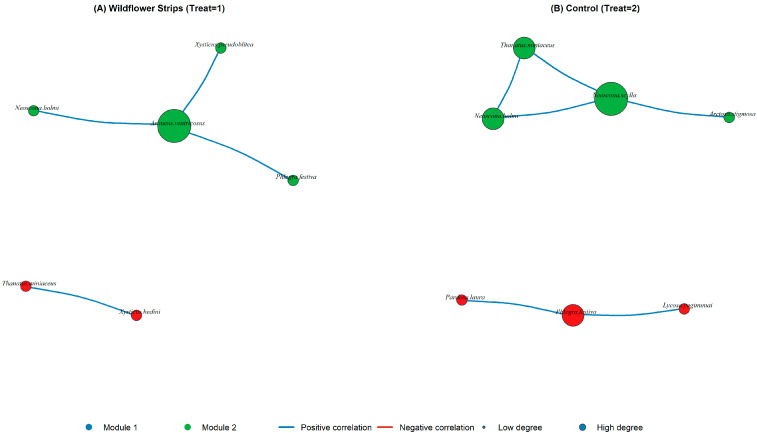
Co-occurrence networks of ground-dwelling spider assemblages in (**A**) Wildflower Strips and (**B**) Control margins. Networks were constructed using pairwise Spearman correlations with stringent rare-species filtering and Benjamini–Hochberg FDR correction (|r| > 0.4, adjusted *p* < 0.05). All edges shown represent statistically significant positive correlations; no significant negative correlations were retained after FDR correction. Node color indicates modular classification (Louvain algorithm; blue = Module 1, green = Module 2), and node size represents the degree (number of connections). The legend includes negative correlation for methodological completeness, though none were retained in the final networks. The networks illustrate a fundamental shift in keystone taxa, with *Neoscona scylla* dominating the control network and *Araneus ventricosus* acting as the central hub in the WFS network.

While the NMDS analysis indicated parallel macro-successional trajectories, co-occurrence network analysis revealed that the internal architecture and species associations were fundamentally restructured by the margin treatment (Figure 9). Both networks were constructed exclusively from significant positive correlations (after FDR correction and rare-species filtering), reflecting co-occurrence patterns structured by shared environmental niches or potential facilitative interactions among the co-occurring taxa. It should be noted that the exclusion of negative correlations from the primary network visualization means that potential competitive or intra-guild predatory interactions are not captured in these network topologies (see Section 4.3 for further discussion). However, there was marked divergence in their topologies.

The control network exhibited a highly clustered and densely interconnected architecture, comprising five distinct modules, with key central nodes (hubs) reaching a maximum degree of 8 (e.g., *Phlegra festiva*). In contrast, the WFS network presented a more decentralized topology with four modules and comparatively lower overall connectivity, with the best connected nodes reaching a maximum degree of 6.

The keystone taxa anchoring these respective networks were entirely distinct, directly linking community architecture to the spatial indicator findings. In the control fields, the dense network core was heavily reliant on hub species *Phlegra festiva* and *Thanatus miniaceus* (the latter of which is a significant interior-associated indicator species, *p* = 0.021). Conversely, in the WFS network, keystone roles shifted to *Araneus ventricosus*, *Xysticus hedini*, and *Xysticus pseudoblitea*. Most strikingly, *X. pseudoblitea*—the strongest edge-avoiding, interior-specialist indicator in the previous analysis (*p* = 0.0005)—was elevated to a critical bridging node in the WFS network.

## 4. Discussion

### 4.1. WFS as Potential Temporal Refuges Buffering Mid-Season Community Decline

The mechanism underlying this refuge effect is likely multifaceted, involving a combination of bottom-up resource provisioning and physical habitat modification. First, the high plant species richness and abundant nectar-producing flora within annual wildflower strips (WFSs) can support diverse arthropod assemblages [12], thereby increasing prey availability for generalist predators like spiders. This “bottom-up” effect is a well-documented driver of predator abundance in conservation biological control [27]. Second, the structural complexity provided by the diverse plant architecture offers a greater variety of microhabitats, including sites for web-building, ambush hunting, and refuge from intraguild predation [12]. This increased habitat complexity can facilitate niche partitioning and support a more diverse predator community. Finally, the dense vegetation likely generates shaded microhabitats with reduced soil-surface temperatures and evapotranspiration, offering a direct physiological buffer against desiccation stress during the hot, dry mid-summer [10,28]. Therefore, the observed temporal buffering effect is likely the integrated result of enhanced trophic resources, improved physical habitat structure, and moderated microclimatic extremes, rather than a consequence of any single factor.

This finding extends beyond the conventional “abundance enhancement” paradigm documented for WFS interventions [12,43,44] in temperate agroecosystems. For instance, Rischen et al. reported that wildflower-sown islands in farmland areas promoted spider diversity primarily through static enrichment of local species pools [16], while Zhang et al. found that flowering fields supported greater spider species richness at field edges than at interiors; however, their assessments were largely cross-sectional [45]. In contrast, the mid-summer buffering we observed in the Yinchuan Plain is more consistent with ecological patterns described in arid-zone studies [46], where microhabitat structures that provide localized thermal and desiccation buffering can be critical to arthropod persistence.

The mechanism underlying this refuge effect likely involves multiple interacting pathways. First, the structural complexity of sown plant communities (Table S1), including *Cosmos bipinnatus*, *Zinnia elegans*, and *Medicago sativa*, is likely to generate shaded microhabitats with reduced soil-surface temperatures and evapotranspiration, though direct in situ measurement is required to confirm this [47,48,49]. Second, the high plant species richness and abundant nectar-producing flora within annual wildflower strips (WFSs) provide favorable microhabitats that sustain diverse arthropod assemblages [7]. Consequently, these strips function as critical source habitats that “export” ground-dwelling spiders and other epigeal predators into the adjacent crop field [50], enhancing biological pest control in areas where the structurally simplified crop matrix offers limited ecological support [51]. The fact that *Lycosa coelestis* exhibited a pronounced population peak within WFS margins specifically during July, while remaining virtually absent in controls, provides taxon-specific evidence consistent with this temporal refuge hypothesis [52]. These dynamics suggest that WFS may be particularly important for maintaining populations of larger, less mobile cursorial hunters that lack the ballooning dispersal capacity of web-building taxa and are therefore more susceptible to localized population declines [53].

The sustained spider abundance and diversity in WFSs during July–August can be directly linked to the staggered flowering phenology of the sown plant assemblage. While natural margins experienced vegetation senescence under peak thermal and hydric stress, the annual components of the WFSs (particularly *Cosmos bipinnatus* and *Zinnia elegans*) maintained active flowering and high nectar production during this critical window. This continuous floral resource availability likely supported diverse prey communities and provided essential carbohydrate sources for cursorial spiders, whose metabolic demands increase under high-temperature conditions [48]. Furthermore, the structural complexity provided by perennial species such as *Medicago sativa* and shrubs at the tomato site offered buffered microhabitats that mitigated desiccation stress. Thus, the temporal buffering effect observed in spider assemblages appears mechanistically driven by the intentional phenological complementarity of the WFS botanical design, which decoupled resource availability from the seasonal decline characteristic of natural ruderal vegetation in arid systems.

### 4.2. Spatial Gradients, Competitive Exclusion, and the Decoupling of Abundance from Diversity

A nuanced spatial pattern emerged from our fine-scale sampling: while overall spider abundance remained relatively stable across the 0–20 m gradient, taxonomic diversity and species richness increased significantly toward the crop interior. This spatial decoupling of abundance and diversity contradicts the intuitive expectation that field margins universally serve as biodiversity hotspots and instead points to strong competitive exclusion and species-specific habitat partitioning at the immediate edge.

The classical edge-effect literature has produced mixed results regarding spider responses to habitat boundaries. Rodrigues et al. [50] found in riparian forests that spider diversity responded strongly to edge effects, sometimes showing reduced diversity at margins due to microclimatic exposure. In agricultural contexts, Plath et al. documented strong edge effects on spider assemblages in European cereal fields, with species richness often declining from boundaries inward [51]. More recently, Kent et al. confirmed significant spatial patterning between field interiors and edges in Canadian canola agroecosystems [52]. However, our findings reveal a qualitatively different pattern: diversity increased toward the interior instead of declining.

A plausible explanation for this inversion is the overwhelming numerical dominance of cursorial Lycosidae at the margin edge. *Pardosa astrigera* and *Arctosa stigmosa* collectively accounted for nearly half of all individuals and exhibited their highest densities in the immediate vicinity of the margin. These mobile, generalist agrobionts likely monopolize resources and exert competitive pressure that suppresses subdominant taxa in the edge zone [53]. In contrast, the crop interior may accommodate a more functionally heterogeneous assemblage, with web-building species (*Neoscona scylla*, *N. holmi*) and ambush-foraging taxa (*Thanatus miniaceus*, *Xysticus pseudoblitea*), as confirmed by our Indicator Species Analysis [54].

This pattern of interior-associated indicator species avoiding competitive margins is consistent with community assembly mechanisms described by Gallé et al., who found that within-field position drives functional diversity of spiders and carabids [55], and by Ferrante et al., who emphasized that habitat characteristics filter for specific traits along landscape gradients [54]. Furthermore, spider species richness peaks at field margins within organically managed agroecosystems—a pattern that is largely attributed to attenuated edge effects [56]—but arid agroecosystems may exhibit pronounced spatial heterogeneity in disturbance intensity and resource availability at boundaries—conditions which are potentially favorable to strong competitive exclusion rather than coexistence [53]. The 20 m interior zone therefore appears to function as a spatially distinct community module, supporting functionally important guilds that contribute disproportionately to pest suppression but are constrained at the edge.

### 4.3. Network Rewiring and the Restructuring of Intra-Guild Interactions

While our NMDS ordination suggested parallel macro-successional trajectories for WFS and control communities, the co-occurrence network analysis revealed that the internal architecture of species associations was fundamentally rewired by margin treatment. The control network exhibited a densely clustered topology with five modules and high interconnectivity centered around *Phlegra festiva* and *Thanatus miniaceus*, whereas the wildflower network was more decentralized, with four modules and keystone roles that shifted toward *Araneus ventricosus*, *Xysticus hedini*, and *X. pseudoblitea*.

This network restructuring has important ecological implications. In ecological network theory, densely clustered architectures with a few dominant hub species are often associated with communities under competitive pressure or resource limitation [57]. The centralized control network may reflect the constrained niche space of natural margins, where a limited number of dominant species monopolize interactions by establishing tight co-occurrence associations with subordinate taxa [58]. In contrast, the more open, decentralized WFS network suggests that the sown plant community expands the available niche space, reducing competitive bottlenecks and allowing a broader suite of species to form associations without relying on a single dominant hub [59]. This shift toward interior-associated specialist taxa (*X. pseudoblitea* in particular) becoming bridging nodes in the WFS network directly mirrors the spatial partitioning identified in our ISA and PERMANOVA results, confirming that sown strips not only alter where species occur but fundamentally restructure how they co-occur and interact.

The positive co-occurrence patterns captured in both networks suggest that the ground-dwelling spider assemblages in this arid system are structured substantially by shared environmental filtering (e.g., tolerance to desiccation, a preference for specific microhabitat structures) and potential facilitative interactions [54,60]. However, we acknowledge an important methodological limitation of our network approach: by focusing on positive correlations, our networks do not capture potential competitive exclusion or intra-guild predation (IGP), which are well-documented structuring forces in spider communities [53,61]. Negative correlations in co-occurrence networks can reveal ecologically critical antagonistic interactions, such as spatial avoidance between dominant cursorial hunters and subordinate web-building taxa, or IGP among lycosid species of different body sizes. The decision to exclude negative correlations from the primary analysis was driven by the high proportion of rare species in our dataset and the associated risk of spurious negative correlations arising from zero-inflated abundance matrices. Future studies with larger sample sizes and higher detection frequencies for subordinate taxa should construct networks incorporating both positive and negative edges to provide a more complete picture of how WFSs restructure the full spectrum of intra-guild interactions—from facilitation to competition—in arid agroecosystems.

This finding is consistent with the observations of Ferrante et al., who emphasized that landscape and habitat characteristics filter for specific spider traits while shaping community assembly patterns [54]. Furthermore, the question of whether WFSs function as “ecological traps” has been raised in European contexts, with Ganser et al. documenting detrimental overwintering effects on carabid beetles and spiders in some sown strip configurations [54]. Our data provide no evidence of an ecological trap scenario: on the contrary, WFSs sustained key populations through the harshest period of the growing season and supported a more decentralized, resilient interaction network. This context-dependent outcome underscores that the ecological function of WFSs is not universal but depends on the regional climate, crop type, and the specific plant species composition of the strip [24,62,63].

### 4.4. Implications for Ecological Pest Control and NbS Design in Arid Landscapes

The spatial and temporal dynamics uncovered here offer actionable insights for deploying Nature-based Solutions in arid and semi-arid agricultural systems, where empirical validations of WFSs remain scarce compared to those of temperate regions. The ability of sown margins to buffer spider population collapse during July–August is particularly significant because this period coincides with peak outbreak windows for many key agricultural pests in the Yinchuan Plain, including aphids (*Aphididae*), mites (*Tetranychidae*), and lepidopteran larvae [64]. Web-building spiders such as Neoscona species are highly effective at capturing the flying adult stages of these pests, while cursorial Lycosidae and Thomisidae provide ground-level predation pressure on soil-dwelling species and those in the early-instar stages [65,66,67]. The fact that WFS sustains both guilds during the critical mid-summer period when control margins experience community collapse suggests that strategically placed wildflower strips could provide continuous biocontrol service delivery at the precise time at which conventional margins fail.

Our spatial findings inform practical strip deployment by demonstrating that a single WFS along a field boundary can influence predator community structure at least 20 m into the crop matrix. The positive edge-to-interior diversity gradient observed within our 0–20 m sampling range indicates that beneficial effects extend well beyond the immediate margin. However, as we did not sample beyond 20 m, it remains unknown whether this trend continues further into large fields. For very large monoculture blocks, the beneficial effects of edge strips may not reach field centers, potentially creating predator-free zones where pest populations can escape top-down control [68]. This supports the recommendation by Tschumi et al. that natural enemy benefits from WFSs are driven by flower availability and proximity, suggesting that distributed “wildflower island” configurations within large fields may be more effective than perimeter-only strips in expansive arid cropping systems [69]. Additionally, the fact that different spider guilds exhibit distinct spatial responses [70] highlights the importance of tailoring WFS plant species composition to support multiple functional groups. Mixed-species seed mixes that combine tall, structurally complex species (for web-building spiders) with low, dense ground cover (for cursorial hunters) are likely to maximize the functional diversity of the predator community [42,47], as suggested by Balzan et al. in their work on augmenting flower trait diversity for multiple ecosystem services [12].

### 4.5. The Critical Role of Context Dependency in NbS Deployment

A crucial insight emerging from our multivariate and site-stratified analyses is the profound context dependency of spider assemblages across the four cropping systems. The NMDS ordination revealed that the overarching community composition was overwhelmingly filtered by site-specific conditions—likely a composite of crop architecture, local microclimate, and regional species pools [68]—rather than by the binary presence of WFS versus control margins. This spatial segregation among wheat, maize, tomato, and apple orchard systems aligns with the “environmental filtering” paradigm in community ecology [60], where local habitat templates dictate the baseline species pool [71] before marginal interventions can exert their effects [68].

Furthermore, the highly heterogeneous responses of both abundance and Shannon diversity across the distance gradients within individual sites (Figure 5B,C) challenge the assumption of a uniform “spillover effect” radiating from ecological infrastructure [11]. In some cropping systems, the margin may act as a robust source habitat [72], while in others, the crop matrix may present insurmountable dispersal barriers [73] or hostile microclimatic conditions for specific guilds [70]. This context dependency carries significant implications for the upscaling of Nature-based Solutions (NbS) in arid landscapes [74,75]. It suggests that standardized, “one-size-fits-all” WFS seed mixes or deployment strategies [76] may yield unpredictable biocontrol outcomes across heterogeneous agricultural mosaics [77]. Instead, precision ecological engineering—where the botanical composition of the WFS is tailored to the specific structural and phenological requirements of the adjacent crop matrix [11] and its associated pest–predator food webs—will be essential to maximize the functional spillover of natural enemies into the crop interior [78].

### 4.6. Limitations and Future Directions

While this study elucidates the spatio-temporal dynamics of spider communities and their response to WFSs in an arid agroecosystem, several limitations should be acknowledged. First, this study did not deploy in situ microclimatic loggers within the wildflower strips or adjacent crop fields. Consequently, while the temporal buffering pattern observed in WFS-assembled spider communities coincides with the period of peak regional thermal and hydric stress, we cannot directly attribute this pattern to localized microclimatic amelioration versus alternative mechanisms such as resource provisioning or structural refuge independent of microclimate. Future studies incorporating fine-scale thermal and humidity profiling within strip and crop microhabitats are essential, as they will allow mechanistic validation of the proposed temporal refuge function [79,80].

Second, the experimental design lacks true independent field replicates within each cropping system. Due to practical constraints in securing multiple commercial fields with identical management histories and paired WFS-control configurations within the same village, our study employed a paired comparative design across four distinct cropping systems rather than a fully replicated block design within a single system. Consequently, the observed differences between WFS and control margins may be partially confounded with site-specific environmental variations, such as local microclimate, crop architecture, and regional species pools. While our multivariate analyses (NMDS and PERMANOVA) explicitly quantified this profound context dependency and demonstrated that site-specific filtering overwhelmingly structured the overarching community composition, the inability to fully disentangle treatment effects from site-level heterogeneity inherently limits the generalizability of the aggregated treatment outcomes. Future studies must prioritize establishing multiple independent field replicates within the same cropping system to rigorously isolate the specific effects of wildflower strips from background environmental noise.

Third, the single-growing-season design inherently limits the assessment of inter-annual variability in spider assemblages. As emphasized by the editor, the patterns observed in 2024 should be interpreted as preliminary insights. Ideally, multi-year monitoring would provide more robust conclusions regarding the temporal stability of the observed refuge effects across different drought and precipitation regimes. However, our meteorological analysis revealed that while 2024 experienced above-average precipitation during the critical sampling months (May–August), temperature conditions remained consistent with historical patterns. Importantly, the positive effects of wildflower strips on spider diversity and biological control potential persisted even under these relatively wetter conditions. This suggests that the observed benefits of wildflower strips may be robust across a range of precipitation regimes, potentially even enhancing their effectiveness in wetter years by supporting greater vegetation complexity and prey diversity. Nevertheless, future long-term studies are warranted to elucidate how inter-annual climatic extremes (e.g., prolonged multi-year droughts) might modulate the biological control potential of wildflower strips and to verify if the “temporal refuge” effect is a consistent phenomenon.

Fourth, while co-occurrence network analysis revealed important shifts in community architecture, it is based on correlation patterns rather than direct behavioral observations of intra-guild interactions [69,81]; future studies combining network analysis with controlled laboratory or field experiments on intra-guild predation and facilitation would provide more mechanistic insights into the observed rewiring [61]. Finally, while our study establishes that WFSs sustain spider populations and restructure community interactions, the direct link between these biodiversity outcomes and measurable reductions in pest damage or crop yield enhancement has yet to be quantified. Integrating pest population monitoring and yield metrics into future WFS trials in the Yinchuan Plain would provide the definitive evidence needed to promote large-scale adoption of wildflower strips as a biocontrol tool in arid agriculture [11,80].

Fifth, our co-occurrence network analysis was limited to positive correlations due to the high prevalence of rare species and zero-inflated data in the spider assemblage. While this approach effectively captured environmentally driven co-occurrence modules, it precluded the detection of potential competitive or intra-guild predatory interactions that would manifest as negative correlations. Although we applied rigorous filtering for rare species and FDR correction for multiple testing, the exclusion of negative edges represents a deliberate analytical trade-off that may oversimplify the true complexity of species interactions. Future network studies in similar arid systems should aim for larger sample sizes to enable the inclusion of negative correlations, thereby providing a more balanced representation of both facilitative and antagonistic community structuring processes.

Sixth, while the botanical composition and intended phenological complementarity of the WFSs are well documented, this study did not include in situ quantitative assessments of flowering intensity, vegetation cover, or biomass dynamics throughout the sampling period. Consequently, our interpretation of the relationship between floral resource persistence and spider responses relies on species-level phenological data from the literature rather than direct field measurements. Future studies should incorporate standardized vegetation monitoring protocols (e.g., Braun-Blanquet method or repeated floral unit counts) alongside arthropod sampling to establish direct, quantitative links between spatiotemporal variation in floral resources and predator community dynamics. Such data would allow for more precise mechanistic modeling of how specific floral traits drive biological control services in arid agroecosystems.

## 5. Conclusions

This study elucidates the spatio-temporal dynamics of ground-dwelling spider assemblages in response to WFSs sown across diverse cropping systems in the arid Yinchuan Plain. Although WFSs did not significantly alter aggregated alpha diversity metrics compared to naturally regenerated margins, they restructured the ecological functioning of the predator community through three interconnected mechanisms.

First, WFSs were associated with sustained high abundance and diversity through periods in which control habitat assemblages exhibited pronounced seasonal decline. This pattern is consistent with the hypothesis that engineered margins may help ensure the persistence of natural enemy communities during windows of elevated environmental stress—though the precise microclimatic mechanisms warrant direct in situ testing in future work.

Second, a significant spatial decoupling of abundance and diversity emerged: while overall abundance remained stable across the 0–20 m gradient due to the overwhelming dominance of cursorial lycosids at the margin edge, taxonomic diversity and species richness increased markedly toward the crop interior. The identification of interior-associated indicator species (*N. scylla*, *N. holmi*, *T. miniaceus*, and *X. pseudoblitea*) confirms that functionally important guilds, including web-building and ambush-foraging taxa, actively avoid competitive edges to colonize the crop interior, where they likely contribute disproportionately to pest suppression.

Third, WFSs were associated with fundamental rewiring of species co-occurrence networks from densely clustered, edge-dependent architectures to more decentralized, open topologies with keystone roles concentrated in interior-colonizing specialist taxa. This network-level restructuring demonstrates that the ecological value of sown margins may extend beyond passive species enrichment to active reorganization of community interaction webs.

Our findings demonstrate that wildflower strips significantly enhance spider diversity and biological control potential in a semi-arid agroecosystem, even under conditions of above-average precipitation. The robust positive effects observed during a relatively wet year (2024) suggest that these conservation measures may provide reliable ecosystem services across a range of climatic conditions. Future research should investigate how wildflower strip design can be optimized to maintain these benefits during extreme drought conditions, which are increasingly common in semi-arid regions due to climate change.

Collectively, these findings challenge the conventional paradigm that ecological infrastructure should be evaluated primarily based on static abundance metrics. In arid agroecosystems, the conservation value of WFS may lie substantially in temporal stabilization and the maintenance of spatial heterogeneity—both of which appear critical to the sustained delivery of biological pest control services.

## Data Availability

The original contributions presented in this study are included in the article/Supplementary Material. Further inquiries can be directed to the corresponding author.

## Supplementary Materials

The following supporting information can be downloaded at https://www.mdpi.com/article/10.3390/insects17070722/s1, Table S1: Agronomic management practices, fertilization, and pesticide application records for the four experimental cropping systems during the 2024 growing season. Table S2: Botanical composition of sown wildflower strips across the four study sites, detailing species identity, sowing proportion, life form, and primary ecological function. Table S3: Taxonomic composition, relative abundance, and occurrence frequency of ground-dwelling spider species in the study area.
