# Supplementary material for: Wildflower Strips Promote Spider Diversity and Biological Control Potential in a Semi-Arid Agroecosystem: Preliminary Insights from a Single Growing Season
Source: Insects. 2026 Jul 13;17(7):722. doi: 10.3390/insects17070722 (PMC13411598; doi:10.3390/insects17070722)
Supplement: Supplementary file 1 [file insects-17-00722-s001.zip › Table S1.pdf]

Table S1. Agronomic management practices, fertilization, and pesticide application records for the four experimental cropping systems during the 2024 growing season.

Site / Crop Type	Field Size (ha)	Crop Variety	Fertilization Regime (N-P_2O_5-K_2O kg/ha)	Irrigation Schedule (Method & Frequency)	Pesticide Applications (Active Ingredient, Rate, and Timing) *
Wuqu Village (Wheat)	2.0	Ningchun 4	Base: 90 kg N, 75 kg P_2O_5, 45 kg K_2O. Top-dressing: 60 kg N at jointing stage.	Surface flood irrigation. 4 times: May 10, Jun 5, Jun 25, Jul 15	1. Imidacloprid, 15 g a.i./ha, May 20<br>2. Lambda-cyhalothrin, 10 g a.i./ha, Jun 15<br>3. Bensulfuron-methyl, 30 g a.i./ha, Apr 25
Pingjipu (Maize)	4.0	Xianyu 335	Base: 120 kg N, 90 kg P_2O_5. Top-dressing: 100 kg N at V6 stage.	Surface flood irrigation. 3 times: May 15, Jun 20, Jul 10	1. Chlorantraniliprole, 30 g a.i./ha, Jul 5<br>2. Atrazine + Acetochlor, 1500 g a.i./ha, May 18
Hongqi Village (Tomato)	1.5	Ningfan 8	Drip fertigation: Total 240 kg N, 180 kg K_2O applied in 8 splits.	Drip irrigation. 12 times, weekly from May to Aug	1. Abamectin, 9 g a.i./ha, Jun 10<br>2. Acetamiprid, 12 g a.i./ha, Jul 20<br>3. Mancozeb, 1500 g a.i./ha, Jun 25
Jinglong Village (Apple Orchard)	2.5	Red Fuji	200 kg N, 150 kg P_2O_5, 200 kg K_2O applied in spring and post-harvest.	Drip irrigation. 8 times, bi-weekly from May to Sep	1. Emamectin benzoate, 15 g a.i./ha, May 25<br>2. Spirodiclofen, 20 g a.i./ha, Jun 18<br>3. Mancozeb, 1200 g a.i./ha, May 10

* **Note:** Pesticide applications listed here refer to the crop field interior. The marginal habitats (both WFS and control margins) were strictly maintained without any synthetic insecticide or herbicide applications throughout the study period.
