# Supplementary material for: Wildflower Strips Promote Spider Diversity and Biological Control Potential in a Semi-Arid Agroecosystem: Preliminary Insights from a Single Growing Season
Source: Insects. 2026 Jul 13;17(7):722. doi: 10.3390/insects17070722 (PMC13411598; doi:10.3390/insects17070722)
Supplement: Supplementary file 1 [file insects-17-00722-s001.zip › Table S2.pdf]

Table S2. Botanical composition of sown wildflower strips across the four study sites, detailing species identity, sowing proportion, life form, and primary ecological function.

Site (Crop Type)	Plant Species	Sowing Proportion	Life Form	Primary Ecological Function
Wuqu Village (Wheat field)	*Calendula officinalis*	20%	Annual	Prolonged nectar/pollen source
	Zinnia elegans	10%	Annual	Nectar source, structural complexity
	Cosmos bipinnatus	10%	Annual	Structural refuge, nectar source
	Centaurea cyanus	10%	Annual	Nectar/pollen source
	Papaver rhoeas	10%	Annual	Early-season pollen source
	Cosmos sulphureus	10%	Annual	Nectar source
	Coreopsis basalis	10%	Annual	Nectar source, ground cover
	Orychophragmus violaceus	10%	Annual	Early-season nectar/pollen source
	Gypsophila paniculata	5%	Perennial	Structural complexity
	Gaillardia pulchella	5%	Annual	Nectar source
Pingjipu (Maize field)	*Astragalus adsurgens*	20%	Perennial	Ground cover, overwintering refuge
	Iris lactea	20%	Perennial	Structural complexity, ground cover
	Coreopsis basalis	10%	Annual	Nectar source, ground cover
	Centaurea cyanus	10%	Annual	Nectar/pollen source
	Orychophragmus violaceus	10%	Annual	Early-season nectar/pollen source
	Zinnia elegans	10%	Annual	Nectar source, structural complexity
	Echinacea purpurea	5%	Perennial	Late-season nectar source
	Medicago sativa	5%	Perennial	Ground cover, nitrogen fixation
	Cosmos bipinnatus	5%	Annual	Structural refuge
	Lavandula angustifolia	5%	Perennial	Nectar source, aromatic pest deterrent
Hongqi Village (Tomato field)	*Melilotus officinalis*	40%	Biennial	Prolonged nectar/pollen, ground cover
	Rudbeckia hirta	10%	Perennial	Late-season nectar source
	Tagetes erecta	10%	Annual	Nectar source, nematode suppression
	Viola philippica	10%	Perennial	Early-season nectar, ground cover
	Sorbaria kirilowii	5%	Shrub	Permanent structural refuge
	Rosa xanthina	5%	Shrub	Permanent structural refuge
	Orychophragmus violaceus	5%	Annual	Early-season nectar/pollen source

**Site (Crop Type)**	**Plant Species**	**Sowing Proportion**	**Life Form**	**Primary Ecological Function**
	Iris lactea	5%	Perennial	Structural complexity
	Papaver rhoeas	5%	Annual	Early-season pollen source
	Cosmos bipinnatus	5%	Annual	Structural refuge
Jinglong Village (Apple orchard)	*Medicago sativa*	100%	Perennial	Ground cover, nitrogen fixation, overwintering habitat
