# Supplementary material for: Wildflower Strips Promote Spider Diversity and Biological Control Potential in a Semi-Arid Agroecosystem: Preliminary Insights from a Single Growing Season
Source: Insects. 2026 Jul 13;17(7):722. doi: 10.3390/insects17070722 (PMC13411598; doi:10.3390/insects17070722)
Supplement: Supplementary file 1 [file insects-17-00722-s001.zip › Table S3.pdf]

**Table S3.** Taxonomic composition, relative abundance, and occurrence frequency of ground-dwelling spider species in the study area

| Family | Species | Treatment (N) | Treatment (%) | Control (N) | Control (%) | Total (N) | Frequency (%) |
|---|---|---|---|---|---|---|---|
| Lycosidae | *Pardosa astrigera* | 375 | 29.62 | 280 | 22.31 | 655 | 73.26 |
| | *Arctosa stigmosa* | 253 | 19.98 | 215 | 17.13 | 468 | 60.76 |
| | *Pardosa laura* | 44 | 3.48 | 47 | 3.75 | 91 | 21.53 |
| | *Lycosa coelestis* | 39 | 3.08 | 50 | 3.98 | 89 | 21.18 |
| | *Lycosa sinesis* | 34 | 2.69 | 47 | 3.75 | 81 | 22.57 |
| | *Lycosa yaginumai* | 42 | 3.32 | 39 | 3.11 | 81 | 23.61 |
| | *Alopecosa licenti* | 41 | 3.24 | 39 | 3.11 | 80 | 22.22 |
| Gnaphosidae | *Gnaphosa kansuensis* | 36 | 2.84 | 42 | 3.35 | 78 | 22.57 |
| | *Drassodes serratidens* | 36 | 2.84 | 42 | 3.35 | 78 | 21.88 |
| | *Gnaphosa sinensis* | 31 | 2.45 | 43 | 3.43 | 74 | 18.75 |
| Thomisidae | *Xysticus striatipes* | 27 | 2.13 | 46 | 3.67 | 73 | 20.14 |
| | *Xysticus pseudoblitea* | 24 | 1.90 | 47 | 3.75 | 71 | 18.06 |
| | *Xysticus hedini* | 47 | 3.71 | 24 | 1.91 | 71 | 17.71 |
| Araneidae | *Neoscona scylla* | 31 | 2.45 | 37 | 2.95 | 68 | 20.14 |
| | *Araneus ventricosus* | 28 | 2.21 | 39 | 3.11 | 67 | 18.75 |
| | *Neoscona holmi* | 33 | 2.61 | 33 | 2.63 | 66 | 16.67 |
| Linyphiidae | *Hylyphantes graminicola* | 29 | 2.29 | 34 | 2.71 | 63 | 15.28 |
| Titanoecidae | *Nurscia albofasciata* | 22 | 1.74 | 36 | 2.87 | 58 | 14.58 |
| Theridiidae | *Enoplognatha margarita* | 28 | 2.21 | 29 | 2.31 | 57 | 15.62 |
| Philodromidae | *Thanatus miniaceus* | 27 | 2.13 | 28 | 2.23 | 55 | 16.32 |
| Salticidae | *Phlegra festiva* | 20 | 1.58 | 32 | 2.55 | 52 | 14.58 |
| Agelenidae | *Agelena labyrinthica* | 19 | 1.50 | 26 | 2.07 | 45 | 14.24 |
